# Relative Perfusion Differences between Parathyroid Adenomas and the Thyroid on Multiphase 4DCT

**DOI:** 10.1155/2022/2984789

**Published:** 2022-05-20

**Authors:** Steven P. M. J. Raeymaeckers, Yannick De Brucker, Maurizio Tosi, Nico Buls, Johan De Mey

**Affiliations:** ^1^Department of Radiology, UZ Brussel, Brussels 1090, Belgium; ^2^Department of Anesthesiology, UZ Brussel, Brussels 1090, Belgium

## Abstract

A multiphase 4DCT technique can be useful for the detection of parathyroid adenomas. Up to 16 different phases can be obtained without significant increase of exposure dose using wide beam axial scanning. This technique also allows for the calculation of perfusion parameters in suspected lesions. We present data on 19 patients with histologically proven parathyroid adenomas. We find a strong correlation between 2 perfusion parameters when comparing parathyroid adenomas and thyroid tissue: parathyroid adenomas show a 55% increase in blood flow (BF) (*p* < 0.001) and a 50% increase in blood volume (BV) (*p* < 0.001) as compared to normal thyroid tissue. The analysis of the ROC curve for the different perfusion parameters demonstrates a significantly high area under the curve for BF and BV, confirming these two perfusion parameters to be a possible discriminating tool to discern between parathyroid adenomas and thyroid tissue. These findings can help to discern parathyroid from thyroid tissue and may aid in the detection of parathyroid adenomas.

## 1. Introduction

Normal-sized parathyroid glands are usually not detectable by imaging modalities [1]. The goal of imaging in patients with primary hyperparathyroidism (PHPT) is the detection of an enlarged parathyroid as a landmark for surgery [2]. Most often, an enlarged parathyroid will prove to be a parathyroid adenoma (80-85% of cases) [1]. In case of multigland disease or parathyroid hyperplasia (15-20%), the sensitivity of imaging is lower: presumably because as more glands are affected, the glands themselves are smaller in size as opposed to a single adenoma [3]. In exceedingly rare cases, PHPT can be caused by a parathyroid carcinoma (<1%) [1].

The concept of 4DCT for the detection of parathyroid adenomas was coined in 2006 by Rodgers et al. [4]. These authors used a three-phasic approach with an unenhanced phase as well as an arterial and venous phase. Many different study protocols have been suggested by different authors over the past 15 years. Most authors favor a triphasic or a 4-phasic approach [5]. Recently, a multiphase 4DCT technique has been described in the literature, by which means up to 16 different phases can be obtained without significant increase of exposure dose. With the addition of more phases, an optimized time window can be found with maximum enhancement of the parathyroid adenoma [6]. This way, the temporary rise in enhancement of the parathyroid during the arterial window can be more readily detected, thus simplifying detection of the lesion.

Since this scanning protocol obtains a nonenhanced scan, multiple arterial scans at a two second delay interval and subsequent venous scans up to 80 s after administration of contrast, it is also possible to calculate perfusion parameters. For a reliable calculation of perfusion maps, a baseline with at least one NECT is required. This way, an accurate baseline subtraction can be performed by the deconvolution algorithm. Also, the image frequency in the arterial phase must be frequent enough to not to miss the arterial peak. This frequency may vary based upon the vascularity of the tissue [7]. For a high blood flow organ like the thyroid/parathyroid, this image interval would have to be less than 3 seconds. Our multiphase 4DCT technique obtains a single NECT and arterial images every 2 seconds for a duration of 20 seconds. This study reports on different perfusion parameters of parathyroid adenomas and the relation of these parameters to the perfusion of the thyroid gland, as differences in enhancement between these two structures are the cornerstone of the identification of parathyroid adenomas on classic 4DCT [8].

## 2. Materials and Methods

We present data on 19 patients with primary hyperparathyroidism (i.e., an elevated serum level of calcium and raised levels of parathyroid hormone). All the patients had undergone a prior US examination, as this is the standard of care for detecting parathyroid adenomas in our hospital. These patients subsequently underwent multiphase 4DCT as a landmark study, prior to surgery. This study was approved by the medical ethics committee of our hospital. All the patients were informed about the nature of the procedure as well as the risks involved (radiation exposure and administration of iodinated contrast) and signed an approved informed consent form prior to the examination.

Patients under the legal age (18 y) were excluded. Patients who had undergone prior surgery of the thyroid or parathyroid were also excluded. Three patients were excluded in this analysis, since no enlarged parathyroid could be detected.

Scanning is performed on a 256-slice Revolution CT (GE Healthcare). The patients receive a venous catheter placed in a cubital vein that is checked for patency. The arms are placed in a neutral position alongside the body. The patient's head is fixed in a head cradle. The sensation of contrast administration is explained to the patients, and they are instructed not to move and to avoid swallowing. Continuous axial scanning centered on the thyroid is performed over a fixed 8 cm coverage volume (100 kVp, SmartmA 10–480 mA, thickness 0.625 mm, 0.5 s rotation scanning time). Wide beam axial scanning was chosen over helical scanning to limit the dose [9]. The scanning range can be increased to a 16 cm volume, to include visualization of the upper mediastinum if an ectopic localization is suspected.

First, we obtain a nonenhanced scan (NECT). Simultaneously with this phase, contrast administration is initiated: a bolus of 90 mL Xenetix 350 mg I/mL is injected at 6 mL/s followed by a 50 mL saline flush (6 mL/s). After a delay of 20 s, 11 subsequent phases with a 2 s interphase delay are obtained (arterial phases). With a 10 s interphase delay, 4 more phases are obtained (venous phases). The effective dose associated with this 4DCT protocol has a mean value of 6.7 mSv and can be as low as 1.4 mSv. The effective dose of 4DCT protocols in the literature is situated between 10.4 mSv and 13.8 mSv [10, 11].

The data were analysed and interpreted by two senior members of the staff with 11 and 12 years of experience in the field of neuroradiology, head and neck radiology, and perfusion imaging experience, respectively.

The images were reviewed on an Advantage Workstation Server 3.2 Ext. 3.4 (GE Healthcare). The different scan phases are deformably registered, after which the registered image data can be analysed on a voxel-by-voxel basis, thereby retaining spatial information for the analysis. Perfusion data is calculated using commercially available perfusion software that allows for modified distributed parameter analysis (CT Perfusion 4D; GE Healthcare). The calculations make use of a model dependent deconvolution algorithm [12]. As “base” noncontrast images, we use the series of images that were acquired before the arrival of the contrast bolus. Regions of interest (ROIs) for computing arterial input (AIF) and venous output (VOF) functions were manually selected. The AIF ROI was placed in the common carotid artery, at the level of the thyroid. The VOF ROI was placed in the internal jugular vein, at the same level of the AIF ROI. Computation of the perfusion maps is based upon the mathematical derivation of an impulse residue function (IRF) from each pixel's time-density function, and the single global AIF, using an algorithm based upon singular value decomposition deconvolution [13, 14]. Delay correction was implemented by model-based estimation of tracer arrival time in each pixel, *t*_0_. Absolute hemodynamic measurements were derived using the venous output function for normalization [15].

The following perfusion parameters were calculated, both for normal thyroid and parathyroid tissue: Blood flow (BF) is defined as the mean rate of blood flow through a tissue [16]. It is expressed in mL/100 g/min. BF was computed as the value of the IRF at *t*_0_. Mean transit time (MTT) is defined as the average time that red blood cells spend within a determinate volume of capillary circulation [16]. It is expressed in seconds (s). MTT was computed as the first moment of the IRF beginning at *t*_0_. Blood volume (BV) is defined as the mean blood volume in a given amount of tissue [16]. It is expressed in mL/100 g. BV was computed as the product of BF and MTT. Tmax is defined as the time to the maximum of the residue function obtained by deconvolution. Tmax represents the arrival delay of the contrast agent between the arterial input function and the tissue, it has proven to surpass delay-corrected parameters such as MTT [17]. It is expressed in seconds (s). Tmax was derived as (MTT ÷ 2) + *t*_0_. The permeability–surface area product (PS) is defined as the flow of molecules through the capillary membranes in a certain volume of tissue [18]. It is expressed in mL/min/100 mL tissue. PS depends not only on the characteristics of the capillary wall but also the contrast agent used. A small or lipophilic contrast agent for instance can pass more easily through the endothelial wall of the capillaries compared to a larger or hydrophilic contrast agent.

ROIs are placed in the enhancing part of suspected parathyroid adenomas as well as in a part of the thyroid that can be considered as normal, based on the 2D images.

An overview of the calculated values for these different perfusion parameters can be found in Table 1. In addition, parametric maps of the perfusion parameters were created with a color-coded overlay.

Statistical analyses were performed using SPSS software ver. 23.0 (IBM, Armonk, NY, USA). For this small dataset, we assumed the absence of normality and symmetry. The Wilcoxon signed rank test was used to evaluate differences between values obtained in the parathyroid adenoma and normal thyroid tissue. Statistical significance was set at *p* < 0.05. In addition, we performed a ROC analysis and calculated the area under the curve (AUC) as a metric of differentiation between perfusion values of parathyroid adenoma and normal thyroid tissue.

## 3. Results

In 3 patients, no enlarged parathyroid adenoma could be detected, either on ultrasound or with 4DCT. These patients did not undergo surgery and were excluded in this study. Three lesions that were detected on 4DCT could not be detected with ultrasound. One was a large intrathoracic and retrotracheal lesion that by no means could have been detected using ultrasound. Two other smaller lesions were missed on ultrasound. All lesions detected on 4DCT could be localised during surgery and were subsequently removed, after which a significant drop in parathyroid hormone levels was observed perioperatively. The resected lesions were sent for anatomopathological analysis, and all lesions were confirmed to be parathyroid adenomas. The average lesion size was 0.96 mL, with the smallest lesion measuring 0.13 mL and the largest lesion measuring up to 5.28 mL.

**Figure 1:**
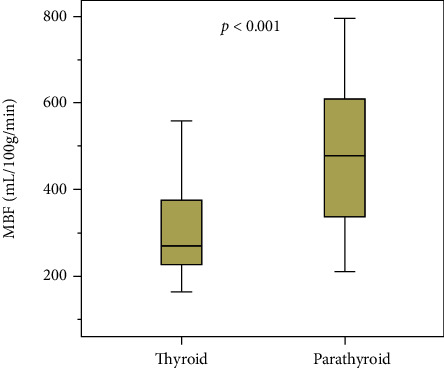
Boxplot. BF normal thyroid tissue vs BF parathyroid adenomas.

**Figure 2:**
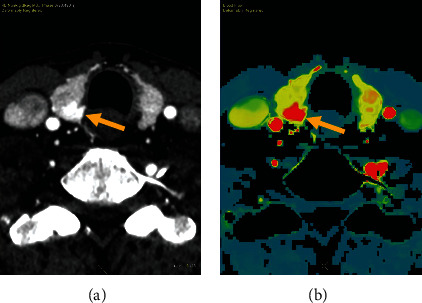
(a) 4DCT. On the arterial phase, we find a large intrathyroidal parathyroid adenoma (arrow). (b) Parametric perfusion map, color-coded overlay of BF values. Note the elevated BF in the parathyroid adenoma as compared to the thyroid tissue.

For all considered perfusion parameters, both BF and BV showed significantly different values in the parathyroid compared to thyroid tissue. BF measured in parathyroid adenomas is 55% higher when compared to normal thyroid tissue (470.4 (St Dev 171.8) vs 304.2 (St Dev 112.6) mL/100 g/min, *p* value <0.001). A boxplot overview of the distribution of these values is provided in Figure 1. An example is provided in Figure 2. In this case, we find a large parathyroid adenoma, located posterior in the right thyroid lobe. On the provided arterial phase, we find an elevated enhancement of the lesion when compared to the thyroid. BF on the color-coded overlay is clearly elevated as well.

**Figure 3:**
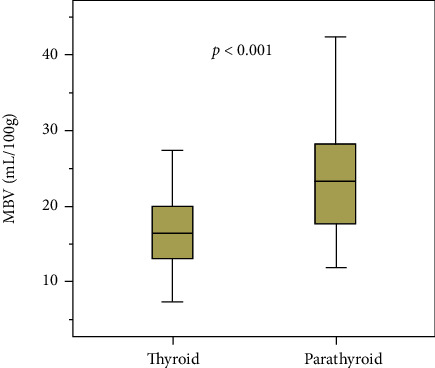
Boxplot. BV normal thyroid tissue vs BV parathyroid adenomas.

**Figure 4:**
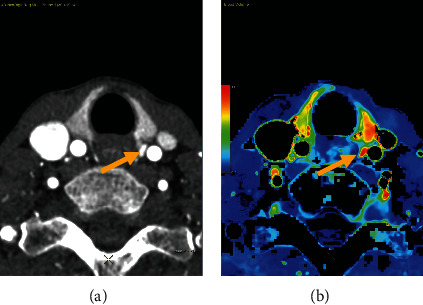
(a) 4DCT. On the arterial phase, we find a small parathyroid adenoma (arrow) in close relation to the left common carotid artery. (b) Parametric perfusion map, color-coded overlay of BV values. Note the elevated BV in the parathyroid adenoma as compared to the thyroid tissue.

BV measured in parathyroid adenomas is 50% higher when compared to normal thyroid tissue (24.1 (St Dev 8.0) vs 16.1 (St Dev 5.5) mL/100 g, *p* value <0.001). A boxplot overview of the distribution of these values is provided in Figure 3. An example is provided in Figure 4. Here, we find a small parathyroid adenoma, located posteriorly in close relation to the left common carotid artery. BV on the color-coded overlay is elevated in comparison to the thyroid.

MTT in parathyroid adenomas and in normal thyroid tissue did not differ significantly (3.7 seconds versus 3.9 seconds). Both higher MTT values are found for parathyroid adenomas as well as lower values when comparing to normal thyroid tissue; this parameter then does not seem relevant. The average Tmax in parathyroid adenomas is lower when compared to normal thyroid tissue (4.9 seconds versus 5.7 seconds), and the average PS is higher in comparison (55.0 s versus 46.8 s). However, for both these perfusion functions, we find the same heterogeneity with higher and lower values in comparison to the thyroid as we do observe for the MTT measurements. No statistical difference can be found.

**Figure 5:**
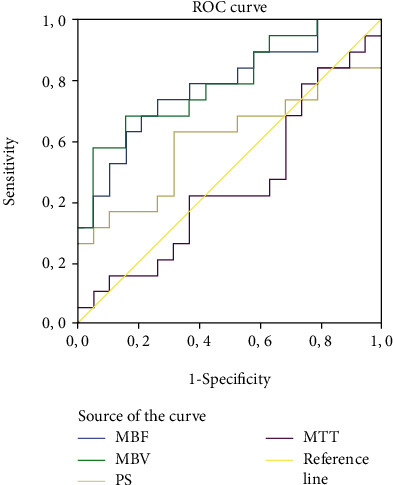
ROC analysis of the perfusion values in parathyroid adenomas compared to thyroid tissue.

The ROC analysis of the perfusion values in parathyroid adenomas compared to thyroid tissue (Figure 5) shows a significant area under the curve (AUC) of 0.78 (St Dev 0.08), *p* = 0.003 and 0.79 (St Dev 0.07), *p* = 0.002 for BF and BV, respectively. The AUCs of MTT (0.61, *p* = 0.651) and PS (0.46, *p* = 0.261) were not significant.

## 4. Discussion

A previous study demonstrated that a multiphase 4DCT protocol may allow for a better visualization of the pattern of enhancement of parathyroid lesions [19]. Enhancement over time curves for suspected lesions can be drawn, in which way wash-in and wash-out of contrast can be readily demonstrated. In this study, we examined different perfusion parameters of parathyroid adenomas and the relation of these parameters to the perfusion of the thyroid gland.

We present data on 19 true positive patients. Three patients were excluded, in which no enlarged parathyroid could be detected on either imaging modality. We do not possess data on the presence of parathyroid adenomas for these negative cases. This study also found no false positive cases. It is not possible to comment on sensitivity or specificity for this study: as normal parathyroid adenomas are very small structures that are usually not visible on imaging, it is also not possible to obtain perfusion values for normal parathyroids.

We find a strong correlation between 2 perfusion parameters when comparing parathyroid adenomas and thyroid tissue: parathyroid adenomas show a 55% increase in BF (*p* < 0.001) and a 50% increase in BV (*p* < 0.001) as compared to normal thyroid tissue. Other perfusion parameters (MTT, PS, and Tmax) did not differ significantly. Also, the analysis of the ROC curve for the different perfusion parameters demonstrates a significantly high area under the curve for BF and BV, confirming these two perfusion parameters to be a possible discriminating tool to discern between parathyroid adenomas and thyroid tissue. The ROC curves for other perfusion parameters (MTT, PS, and Tmax) do not differ significantly from the null hypothesis.

In this study, all lesions in suspected anatomical positions with an increase in BF and BV (as compared to normal thyroid tissue) were proven to be parathyroid adenomas. We would suspect a similar behaviour in case of multigland disease; however, we do not possess data to corroborate this statement. As more glands are affected, the glands themselves may be smaller in size as opposed to a single adenoma and thus more difficult to detect [3]. A parathyroid carcinoma could also be considered in the differential diagnosis of PHPT; this disease is however also increasingly rare (reported incidence 0.5 to 5% of cases) [20]. Again, no such case was found.

Known mimickers of parathyroid adenomas exist in the form of ectopic thyroid tissue and cervical lymph nodes. It can be suggested that ectopic thyroid tissue would behave in a similar way to normal thyroid tissue and thus present with significantly lower BF and BV as opposed to parathyroid adenomas. Cervical lymph nodes are another known mimicker of parathyroid adenomas. We would not expect to find a significantly higher BF and BV for these nodes as compared to the thyroid, except perhaps in the case of cervical lymph node metastasis. In metastasized nodes, we could expect to find elevated BF and/or BV values. In a patient presenting with PHPT symptoms however, lymph node metastasis would have to be considered an incidentaloma and thus proven to be rare.

Following limitations merit consideration. Quantitative perfusion measurements are complicated by movement [21]. Furthermore, a large variability of absolute perfusion values can be found for different organs (e.g., brain [22]) in both normal subjects and clinical patients. By instructing the patient not to move and to avoid swallowing, we can minimize movement. Also, errors due to movement can be further reduced by performing deformable registration of the time series. The variability of the different perfusion values is less problematic in our specific study, as we do not consider absolute perfusion values but rather the relative difference in enhancement between two related structures (parathyroid and thyroid tissue) within the same patient.

For a reliable calculation of perfusion maps, some considerations have to be met. A reliable baseline with at least one NECT is required. This way, an accurate baseline subtraction can be performed by the deconvolution algorithm. Our multiphase 4DCT technique obtains a single NECT, which is adequate. Second, the image frequency in the arterial phase must be frequent enough to not to miss the arterial peak. Our protocol obtains arterial images every 2 seconds for a duration of 20 seconds and can then be used to calculate perfusion maps. For the calculation of vessel PS product however, the time density curve must be sampled beyond the traditional venous phase: the acquisition interval should be longer than 90 s [7]. This last condition is not met in our study, as our acquisition interval is limited to 80 s. In consequence, the reported PS values in this study should be considered with scrutiny.

This study is also prone to selection bias: we only included patients with positive findings prior to surgery. Lesions in patients when surgery is not considered may be smaller, which may hinder detection and effective calculation of perfusion parameters.

Comparing the suspected parathyroid lesion to thyroid tissue may also prove problematic in cases of thyroid disease such as thyroiditis: here, associated inflammation of the thyroid gland may yield abnormal perfusion of the gland. This consideration may also prove relevant in case of thyroid nodules, a prevalent condition: one could hypothesize that some of these lesions might be mistaken for an enlarged intrathyroidal parathyroid adenoma based on abnormal perfusion values. Thyroid cancer, lastly, is a less prevalent condition as compared to benign thyroid nodules but could also be associated with elevated BF/BV values.

## 5. Conclusions

Despite the small sample size of the study, we find a strong correlation between 2 perfusion parameters when comparing parathyroid adenomas and thyroid tissue: parathyroid adenomas show a 55% increase in BF and a 50% increase in BV as compared to normal thyroid tissue. The analysis of the ROC curve for the different perfusion parameters demonstrates a significantly high area under the curve for BF and BV, confirming these two perfusion parameters to be a possible discriminating tool to discern between parathyroid adenomas and thyroid tissue. These findings may aid in the detection of parathyroid adenomas.

## Figures and Tables

**Table 1:** Overview of the different perfusion values of parathyroid adenomas vs normal thyroid tissue: Blood Flow (BF), Blood Volume (BV), Mean Transit Time (MTT), Tmax, Permeability–Surface area product (PS). (^∗^*p* < 0.001).

Case	BF parathyroid (mL/100 g /min)	BF thyroid (mL/100 g/ min)	BV parathyroid (100 g/ min)	BV thyroid (100 g/ min)	MTT parathyroid (s)	MTT thyroid (s)	Tmax parathyroid (s)	Tmax thyroid (s)	PS parathyroid (mL/min/ 100 mL)	PS thyroid (mL/min/ 100 mL)
1	391.0	270.1	42.4	16.7	6.6	4.4	4.1	5.1	96.1	30.6
2	491.8	257.2	18.4	9.5	2.3	2.2	4.3	5.4	113.9	65.9
3	795.7	460.7	26.4	16.5	1.9	2.2	3.9	6.4	78.7	35.0
4	601.4	500.0	31.9	19.4	2.5	2.7	4.7	7.0	15.9	27.6
5	437.5	286.5	15.4	13.6	2.2	3.0	5.7	6.0	31.5	42.8
6	479.2	261.2	17.3	8.8	2.4	2.0	6.9	8.4	55.6	36.7
7	261.0	210.4	12.0	7.6	3.9	2.7	5.2	8.6	37.0	41.8
8	689.6	413.2	26.0	17.4	2.3	2.6	3.7	5.0	58.1	52.5
9	522.4	236.0	37.7	22.5	4.8	6.0	5.6	4.1	56.3	59.1
10	211.2	194.0	31.9	27.4	9.3	8.7	3.4	2.9	62.8	41.5
11	558.1	399.2	22.2	20.1	2.6	3.2	5.0	4.5	76.1	19.9
12	620.7	559.6	21.0	20.2	2.0	2.2	3.2	4.7	34.2	53.0
13	618.8	276.7	24.8	22.5	2.5	5.0	5.3	3.9	79.9	21.6
14	406.4	283.4	14.6	12.7	2.2	3.0	6.9	5.6	57.0	57.1
15	285.5	186.8	14.0	14.0	3.3	7.2	6.0	6.3	12.5	52.8
16	423.4	245.5	23.3	19.9	3.6	6.0	6.0	4.6	52.5	61.5
17	212.8	164.3	28.0	15.8	8.6	5.8	6.9	5.7	53.6	53.5
18	266.3	219.2	23.2	7.4	5.4	2.1	1.4	6.2	2.7	74.6
19	665.0	356.4	28.4	13.7	2.6	2.3	5.1	7.2	69.9	61.4
Mean	470.4^∗^	304.2	24.1^∗^	16.1	3.7	3.9	4.9	5.7	55.0	46.8
St Dev	171.8	112.6	8.0	5.5	2.2	2.0	1.4	1.5	28.4	15.4

## Data Availability

The statistical data used to support the findings of this study are available from the corresponding author upon request. The full sets of medical images used to support the findings of this study have not been made available because of patient confidentiality.
